# Application of the Higher-Order Hamilton Approach to the Nonlinear Free Vibrations Analysis of Porous FG Nano-Beams in a Hygrothermal Environment Based on a Local/Nonlocal Stress Gradient Model of Elasticity

**DOI:** 10.3390/nano12122098

**Published:** 2022-06-17

**Authors:** Rosa Penna, Luciano Feo, Giuseppe Lovisi, Francesco Fabbrocino

**Affiliations:** 1 Department of Civil Engineering, University of Salerno, 84084 Fisciano, Italy; lfeo@unisa.it (L.F.); glovisi@unisa.it (G.L.); 2 Department of Engineering, Pegaso Telematic University, 80143 Naples, Italy; francesco.fabbrocino@unipegaso.it

**Keywords:** porous functionally graded materials, nanobeams, vibrations, local/nonlocal stress gradient elasticity, hygro-thermal loads, higher-order Hamiltonian approach, nonlinear oscillator, Galërkin method

## Abstract

Nonlinear transverse free vibrations of porous functionally-graded (FG) Bernoulli–Euler nanobeams in hygrothermal environments through the local/nonlocal stress gradient theory of elasticity were studied. By using the Galerkin method, the governing equations were reduced to a nonlinear ordinary differential equation. The closed form analytical solution of the nonlinear natural flexural frequency was then established using the higher-order Hamiltonian approach to nonlinear oscillators. A numerical investigation was developed to analyze the influence of different parameters both on the thermo-elastic material properties and the structural response, such as material gradient index, porosity volume fraction, nonlocal parameter, gradient length parameter, mixture parameter, and the amplitude of the nonlinear oscillator on the nonlinear flexural vibrations of metal–ceramic FG porous Bernoulli–Euler nano-beams.

## 1. Introduction

Nanostructures made of temperature-dependent functionally graded materials (FGMs) have played a key role in the advancement of nanotechnologies for the design of devices such as nanoswitches, nanosensors, nanoactuators, and nanogenerators, as well as nanoelectromechanical systems (NEMS), for use even under extreme temperature and humidity conditions [1,2,3,4,5,6,7,8]. Recent studies have also shown that, by managing some fabrication parameters during the manufacture of FGMs, different kinds of porosity distributions can be obtained within their structure to further improve the physical and mechanical characteristics of the material [9,10,11,12,13,14,15,16,17,18,19].

Therefore, it is necessary to research theoretical models that can capture the small effects in the overall mechanical response of the porous FG structure and the hygrothermal ones that cause damage due to the expansion of the material and the initial stresses induced by the hygrothermal conditions. It is well-known that the size-dependent behavior of nanostructures, observed in experimental nanoscale tests and atomistic simulations [20], cannot be captured by the classical constitutive law that does not include size effects. In order to overcome the complexity of the experimental tests at nanoscale and the high computational cost of the atomistic simulations, several higher-order continuum mechanics theories have been developed in the last years. The two milestones on this topic are Eringen’s strain-driven nonlocal integral model (Eringen’s StrainDM) [21,22] and Lim’s nonlocal strain gradient theory (Lim’s NStrainGT) [23], which have been widely used in a large number of investigations, respectively, in [24,25,26,27,28,29] and [30,31,32,33,34,35], due to their simply differential formulation.

As widely argued in [36] for Eringen’s StrainDM and in [37] for Lim’s NStrainGT, both theories have been declared ill-posed since the constitutive boundary conditions are in conflict with equilibrium requirements. Their inapplicability was bypassed by using other theories such as the local-nonlocal mixture constitutive model [38], the coupled theories [39], or resorting the stress-driven nonlocal integral model (StressDM) [40]. More recently, based on a variational approach, the local/nonlocal strain-driven gradient (L/NStrainG) and local/nonlocal stress-driven gradient (L/NStressG) theories were used by Romano and Sciarra in [41,42] to examine the size-dependent structural problems of nano-beams via a mathematically and mechanically consistent approach.

Although several studies were used to assess small effects both in the static and dynamic behavior, as well as in the buckling response of a nanobeam in hygrothermal environments, to the best of the authors’ knowledge the research on the mechanical behavior of nanobeams in extreme conditions is not sufficient. In order to help fill some knowledge gaps on this topic, based on the nonlocal elasticity theory, the hygrothermal static behavior [43] and the vibration and buckling response of an FG sandwich nanobeam were analyzed in [44].

Recent studies were developed using innovative L/NStrainG and/or the L/NstressG theories. In detail, the bending response and the free linear vibration of porous FG nanobeams under hygrothermal environments were analyzed by the same authors of this paper in [45,46]. Moreover, the dynamic response of Bernoulli–Euler multilayered polymer functionally graded carbon nanotubes-reinforced composite nano-beams subjected to hygro-thermal environments was investigated in [47]. In addition, in [48], the L/NStrainG theory was adopted to study the effect of a hygrothermal environment on the buckling behavior of 2D FG Timoshenko nanobeams.

The main aim of this study is to help fill these gaps by proposing an application of the higher-order Hamilton approach [49,50,51,52,53,54,55,56,57] to the nonlinear free vibrations analysis of porous FG nano-beams in a hygro-thermal environment based on the L/NStressG model. 

In particular, the nonlinear transverse free vibrations of a Bernoulli–Euler nano-beam made of a metal–ceramic functionally graded porous material in a hygrothermal environment, with von Kármán type nonlinearity were studied employing the local/nonlocal stress-driven integral model. By using the Galerkin method, the governing equations were reduced to a nonlinear ordinary differential equation. The closed form analytical solution of the nonlinear natural flexural frequency was then established using the higher-order Hamiltonian approach to nonlinear oscillators.

Finally, a numerical investigation was developed to analyze the influence of different parameters both on the thermo-elastic material properties and the structural response, such as material gradient index, porosity volume fraction, nonlocal parameter, gradient length parameter, mixture parameter, and the amplitude of nonlinear oscillator on the nonlinear flexural vibrations of metal–ceramic FG porous Bernoulli–Euler nano-beams.

## 2. Functionally Graded Materials

**Figure 1:**
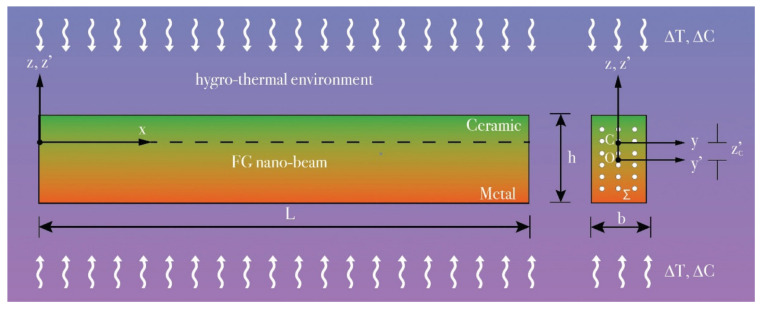
Coordinate system and configuration of a porous FG Bernoulli–Euler nano-beam.

Considering a porous functionally graded (FG) nano-beam with length “L” made of a ceramic (Si_3_N_4_)/metal (SuS_3_O_4_) material and subjected to hygrothermal loadings as shown in Figure 1, in which *y’* and *z’* are the principal axes of the geometric inertia originating at the geometric center O of its rectangular cross-section, Σ(*x*), having thickness “h” and width “b”.

As already shown in [46], the effective value of the FG material generic property,$\;f\left(z^{\prime }\right),\;$can be obtained as a combination of the corresponding thermo-elastic and physical properties of ceramic,$\;f_{c}$, and metal, $f_{m}$, by using the following rule of mixture equation

(1) $$f\left(z^{\prime }\right)=f_{m}+\left(f_{c}-f_{m}\right){\left(\frac{1}{2}+\frac{z^{\prime }}{h}\right)}^{k}-\frac{\zeta }{2}\left(f_{c}+f_{m}\right)\;$$

where *k* (*k* ≥ 0) and *ζ* (*ζ* << 1) are the gradient index and the porosity volume fraction of the FG material, respectively. 

The characteristic values$,\;P_{0},\;$of the thermo-elastic properties of the two constituent materials, in terms of the Young’s modulus, *E_c_* and *E_m_*, mass density, *ρ_c_* and *ρ_m_*, thermal expansion coefficient, *α_c_* and *α_m_*, and moisture expansion coefficient, *β_c_* and *β_m_*, are summarized in the following Table 1.

It is well-known that the temperature dependence of the generic elastic property, $P=P\left(T\right),$ is taken into account with the following nonlinear expression:

(2) $$P\left(T\right)=P_{0}\left(1+X_{-1}\;T^{-1}+X_{1}\;T+X_{2}\;T^{2}+X_{3}\;T^{3}\right)$$

being $X_{-1},X_{1},X_{2},\;$and $X_{3}\;$the coefficients of the material phases for ceramic and metal (Table 2).

Moreover, by evaluating the thermo-elastic material properties with respect to the elastic Cartesian coordinate system (Figure 1), originating at the elastic center *C*, whose position, $z_{c}^{\prime },\;$is expressed as

(3) $$z_{c}^{\prime }=\frac{\int_{\Sigma }E\left(z^{\prime },T\right)z^{\prime }d\Sigma \;}{\int_{\Sigma }E\left(z^{\prime },T\right)d\Sigma \;}\;$$

the bending–extension coupling, due to the variation of the functionally graded material, is eliminated.

## 3. Governing Equations

Under the assumption of Bernoulli–Euler beam theory, the only nonzero Cartesian components of the displacement field can be expressed by

(4) $$u_{x}\left(x,z,t\right)=u\left(x,t\right)-z\frac{\partial w}{\partial x}\left(x,t\right)\;$$

(5) $$u_{z}\left(x,z,t\right)=w\left(x,t\right)\;$$

being $u_{x}\left(x,z,t\right)$,$\;u_{z}\left(x,z,t\right)$ the displacement components along *x* and *z* directions, and *u(*x,t*), w(*x,t*)* the axial and transverse displacements of the elastic centre *C*, at time *t*, respectively.

According to conventional Von-Kármán geometrical nonlinearity, which includes small strains but moderately large rotation, the elastic axial strain is given as

(6) $$\varepsilon_{xx}=\varepsilon_{xx}\left(x,t\right)=\frac{\partial u}{\partial x}+\frac{1}{2}{\left(\frac{\partial w}{\partial x}\right)}^{2}-z\frac{\partial^{2}w}{\partial x^{2}}=\varepsilon^{\left(vK\right)}-z\;\chi \;$$

where the “Von-Kármán” strain, $\;\varepsilon^{\left(vK\right)}$, and the geometrical curvature, χ, have the following expressions

(7) $$\;\varepsilon^{\left(vK\right)}=\frac{\partial u}{\partial x}+\frac{1}{2}{\left(\frac{\partial w}{\partial x}\right)}^{2}\;$$

(8) $$\chi =\frac{\partial^{2}w}{\partial x^{2}}$$

In the case of free vibrations, the nonlinear equations of motion are derived by using the Hamilton’s principle

(9) $$\frac{\partial N\left(x,t\right)}{\partial x}=A_{\rho }\frac{\partial^{2}u\left(x,t\right)}{\partial t^{2}}\;$$

(10) $$\frac{\partial^{2}M}{\partial x^{2}}+\frac{\partial }{\partial x}\left(N\frac{\partial w}{\partial x}\right)-\left(N^{T}+N^{C}\right)\frac{\partial^{2}w}{\partial x^{2}}=A_{\rho }\frac{\partial^{2}w}{\partial t^{2}}-I_{\rho }\;\frac{\partial^{4}w}{\partial x^{2}\;\partial t^{2}}$$

with the corresponding boundary conditions at the nano-beam ends:

(11) $$u\left(x,t\right)\;\;\;\mathrm{or}\;\;\;N\left(x,t\right)$$

(12) $$-\frac{\partial w\left(x,t\right)}{\partial x}\;\mathrm{or}\;\;\;M\left(x,t\right)$$

(13) $$w\left(x,t\right)\;\;\mathrm{or}\;\;\;V\left(x,t\right)=\frac{\partial M\left(x,t\right)}{\partial x}-\left(N^{T}+N^{C}\right)\frac{\partial w\left(x,t\right)}{\partial x}$$

where $N\left(x,t\right)$, $M\left(x,t\right)$, and $V\left(x,t\right)\;$denote the local axial force, the bending moment resultant and the equivalent shear force, respectively. In Equations (9) and (10), $I_{\rho }$ and $A_{\rho }$ are, respectively, the temperature-dependent rotary inertia and the effective cross-sectional mass of the porous FG nano-beam, expressed as follows

(14) $$I_{\rho }=b\int_{-\frac{h}{2}-z_{c}^{\prime }}^{\frac{h}{2}-z_{c}^{\prime }}\rho \left(z,T\right)z^{2}dz$$

(15) $$A_{\rho }=b\int_{-\frac{h}{2}-z_{c}^{\prime }}^{\frac{h}{2}-z_{c}^{\prime }}\rho \left(z,T\right)dz$$

and $N^{T}\;$and $N^{C}\;$denote the hygro-thermal axial force resultants, respectively, defined as

(16) $$N^{T}=N^{T}\left(z,T\right)=\underset{\Sigma }{\int }E\left(z,T\right)\;\alpha \left(z,T\right)\;\Delta Tdz$$

(17) $$N^{C}=N^{C}\left(z,T\right)=\underset{\Sigma }{\int }E\left(z,T\right)\;\beta \left(z,T\right)\;\Delta Cdz$$

in which ΔT and ΔC are the increments of the temperature and moisture concentration, respectively. In the following, we will also denote $E\left(z,T\right)=E.$

## 4. Local/Nonlocal Stress Gradient (NStressG) Model of Elasticity

As shown in [46], by using the local/nonlocal stress gradient integral formulation, the elastic axial strain,${\;\varepsilon }_{xx}$, can be expressed by the following constitutive mixture equation

(18) $$\varepsilon_{xx}=\xi_{1}\frac{\sigma_{xx}\left(x\right)}{E}+\frac{\left(1-\xi_{1}\right)}{E}\;\int_{0}^{L}\Phi_{L_{c}}\left(x-\xi ,L_{c}\;\right)\;\sigma_{xx}\left(\xi \right)d\xi -\frac{1}{E}L_{l}^{2}\;\frac{\partial }{\partial x}\int_{0}^{L}\Phi_{L_{c}}\left(x-\xi ,\;L_{c}\;\right)\;\frac{\partial \sigma_{xx}\left(\xi \right)}{\partial x}d\xi \;$$

being: x and ξ the position vectors of the points of the domain at time t*;* $\sigma_{xx}\;$and $\frac{\partial \sigma_{xx}\;}{\partial_{x}}$ the axial stress component and its gradient, respectively;$\;\xi_{1}\;$the mixture parameter; $\Phi_{L_{c}}\;$the scalar averaging kernel$;L_{c}\;$and $L_{l}\;$the length-scale and the gradient length parameters, respectively.

By choosing the bi-exponential function for the kernel $\Phi_{L_{c}}$ as

(19) $$\Phi_{L_{c}}\left(x,\;L_{c}\right)=\frac{1}{2L_{c}}\exp\;(-\frac{\left|x\right|}{L_{c}}\;{)}_{\;}$$

the integro-differential relation of Equation (18) admits the following solution

(20) $$\varepsilon_{xx}-L_{c}^{2}\;\frac{\partial^{2}\varepsilon_{xx}}{\partial x^{2}}=\frac{\sigma_{xx}}{E}-\frac{L_{c}^{2}}{E}\left(\xi_{1}+\frac{L_{l}^{2}}{L_{c}^{2}}\right)\frac{\;\partial^{2}\sigma_{xx}}{\partial x^{2}}$$

with x ∈ [0, L], if and only if the following two pairs of constitutive boundary conditions (CBCs) are satisfied at the nano-beam ends

(21) $$\left\{\begin{array}{c} \frac{\partial \varepsilon_{xx}\left(0\right)}{\partial x}-\frac{1}{L_{c}}\varepsilon_{xx}\left(0\right)=-\frac{1}{E}\frac{\xi_{1}}{L_{c}}\sigma_{xx}\left(0\right)+\frac{1}{E}\;\left(\xi_{1}+\frac{L_{l}^{2}}{L_{c}^{2}}\right)\;\frac{\partial \sigma_{xx}\left(0\right)}{\partial x}\\ \frac{\partial \varepsilon_{xx}\left(L\right)}{\partial x}+\frac{1}{L_{c}}\varepsilon_{xx}\left(L\right)=\frac{1}{E}\frac{\xi_{1}}{L_{c}}\sigma_{xx}\left(L\right)+\frac{1}{E}\;\left(\xi_{1}+\frac{L_{l}^{2}}{L_{c}^{2}}\right)\;\frac{\partial \sigma_{xx}\left(L\right)}{\partial x} \end{array}\right.$$

## 5. Nonlinear Transverse Free Vibrations (NStressG)

Following the mathematical derivations summarized in Appendix A, we obtain the nonlinear transverse free vibrations equation based on a local/nonlocal stress gradient model of elasticity

(22) $$-I_{E}\;\frac{\partial^{4}w\left(x,t\right)}{\partial x^{4}}+I_{E}L_{c}^{2}\frac{\partial^{6}w\left(x,t\right)}{\partial x^{6}}+L_{c}^{2}\left(\xi_{1}+\frac{L_{l}^{2}}{L_{c}^{2}}\right)\frac{\partial^{2}}{\partial x^{2}}\left(A_{\rho }\frac{\partial^{2}w\left(x,t\right)}{\partial t^{2}}-I_{\rho }\;\frac{\partial^{4}w\left(x,t\right)}{\partial x^{2}\;\partial t^{2}}-\frac{\partial }{\partial x}\left(\left(\frac{A_{E}}{L}\;\int_{0}^{L}\left(\frac{1}{2}{\left(\frac{\partial w\left(x,t\right)}{\partial x}\right)}^{2}-\;L_{c}^{2}\;\frac{\partial^{2}}{\partial x^{2}}\;\left(\frac{1}{2}{\left(\frac{\partial w\left(x,t\right)}{\partial x}\right)}^{2}\right)\right)dx\right)\frac{\partial w\left(x,t\right)}{\partial x}\right)+\left(N^{T}+N^{C}\right)\frac{\partial^{2}w\left(x,t\right)}{\partial x^{2}}\right)=\left(A_{\rho }\frac{\partial^{2}w\left(x,t\right)}{\partial t^{2}}-I_{\rho }\;\frac{\partial^{4}w\left(x,t\right)}{\partial x^{2}\;\partial t^{2}}-\;\frac{\partial }{\partial x}\left(\left(\frac{A_{E}}{L}\;\int_{0}^{L}\left(\frac{1}{2}{\left(\frac{\partial w\left(x,t\right)}{\partial x}\right)}^{2}-L_{c}^{2}\;\frac{\partial^{2}}{\partial x^{2}}\;\left(\frac{1}{2}{\left(\frac{\partial w\left(x,t\right)}{\partial x}\right)}^{2}\right)\right)dx\right)\frac{\partial w\left(x,t\right)}{\partial x}\right)+\left(N^{T}+N^{C}\right)\frac{\partial^{2}w\left(x,t\right)}{\partial x^{2}}\right)$$

By introducing the following dimensionless quantities

(23) $$\tilde{x\;}=\frac{x}{L};\tilde{w}\left(\tilde{x},t\right)=\frac{w\left(x,t\right)}{L};\;\lambda_{c}=\frac{L_{c}}{L}\;;\;\;\lambda_{l}=\frac{L_{l}}{L}\;\;;\;\;\tilde{A_{\rho }}=\frac{A_{\rho }L^{4}}{I_{E}}\;;\;{\tilde{g}}^{2}=\frac{1}{L^{2}}\frac{I_{\rho }}{\tilde{A_{\rho }}};\;\;{\tilde{r}}^{2}=\frac{L^{2}A_{E}}{I_{E}};\;\tilde{N^{T}}=\frac{L^{2}}{I_{E}}N^{T};\tilde{N^{C}}=\frac{L^{2}}{I_{E}}N^{C};\;\tilde{N}=\frac{L^{2}}{I_{E}}\hat{N};\;{\tilde{\omega }}^{2}=\omega^{2}\tilde{A_{\rho }}\;$$

in which $A_{E}$ and $I_{E}$ are the axial and bending stiffnesses of an FG nano-beam, respectively, defined as

(24) $$I_{E}=b\int_{-\frac{h}{2}-z_{c}^{\prime }}^{\frac{h}{2}-z_{c}^{\prime }}E\left(z,T\right)z^{2}dz$$

(25) $$A_{E}=b\int_{-\frac{h}{2}-z_{c}^{\prime }}^{\frac{h}{2}-z_{c}^{\prime }}E\left(z,T\right)dz$$

Equation (22) can be rewritten as

(26) $$\begin{array}{cc} -\frac{\partial^{4}\tilde{w}\left(\tilde{x},t\right)}{\partial {\tilde{x}}^{4}}+\lambda_{c}^{2}\; & \frac{\partial^{6}\tilde{w}\left(\tilde{x},t\right)}{\partial {\tilde{x}}^{6}}+\tilde{A_{\rho }}\lambda_{c}^{2}\left(\xi_{1}+\frac{\lambda_{l}^{2}}{\lambda_{c}^{2}}\right)\left(\frac{\partial^{4}\tilde{w}\left(\tilde{x},t\right)}{\partial {\tilde{x}}^{2}\partial t^{2}}-{\tilde{g}}^{2}\frac{\partial^{6}\tilde{w}\left(\tilde{x},t\right)}{\partial {\tilde{x}}^{4}\partial t^{2}}\right)\\ & -\lambda_{c}^{2}\left(\xi_{1}+\frac{\lambda_{l}^{2}}{\lambda_{c}^{2}}\right)\left({\tilde{r}}^{2}\frac{\partial^{3}}{\partial {\tilde{x}}^{3}}\left(\tilde{N}\frac{\partial \tilde{w}\left(\tilde{x},t\right)}{\partial \tilde{x}}\right)-\left(\tilde{N^{T}}+\tilde{N^{C}}\right)\frac{\partial^{4}\tilde{w}\left(\tilde{x},t\right)}{\partial {\tilde{x}}^{4}}\right)\\ & =\tilde{A_{\rho }}\left(\frac{\partial^{2}\tilde{w}\left(\tilde{x},t\right)}{\partial t^{2}}-{\tilde{g}}^{2}\frac{\partial^{4}\tilde{w}\left(\tilde{x},t\right)}{\partial {\tilde{x}}^{2}\partial t^{2}}\right)\\ & -\left({\tilde{r}}^{2}\frac{\partial }{\partial \tilde{x}}\left(\tilde{N}\frac{\partial \tilde{w}\left(\tilde{x},t\right)}{\partial \tilde{x}}\right)-\left(\tilde{N^{T}}+\tilde{N^{C}}\right)\frac{\partial^{2}\tilde{w}\left(\tilde{x},t\right)}{{\tilde{x}}^{2}}\right) \end{array}$$

Finally, by imposing the dimensionless term ${\tilde{r}}^{2}$ equal to zero, on which the nonlinear nature of the equations depends, from the previous equation, we obtain the linear transverse free oscillations equation

(27) $$\begin{array}{cc} \lambda_{c}^{2}\;\frac{\partial^{6}\tilde{w}\left(\tilde{x},t\right)}{\partial {\tilde{x}}^{6}} & -\frac{\partial^{4}\tilde{w}\left(\tilde{x},t\right)}{\partial {\tilde{x}}^{4}}+\lambda_{c}^{2}\left(\xi_{1}+\frac{\lambda_{l}^{2}}{\lambda_{c}^{2}}\right)\left(\left(\tilde{N^{T}}+\tilde{N^{C}}\right)\frac{\partial^{4}\tilde{w}\left(\tilde{x},t\right)}{\partial {\tilde{x}}^{4}}\right)-\left(\left(\tilde{N^{T}}+\tilde{N^{C}}\right)\frac{\partial^{2}\tilde{w}\left(\tilde{x},t\right)}{{\tilde{x}}^{2}}\right)\\ & =\tilde{A_{\rho }}\left(\frac{\partial^{2}\tilde{w}\left(\tilde{x},t\right)}{\partial t^{2}}-{\tilde{g}}^{2}\frac{\partial^{4}\tilde{w}\left(\tilde{x},t\right)}{\partial {\tilde{x}}^{2}\partial t^{2}}\right)\\ & -\tilde{A_{\rho }}\lambda_{c}^{2}\left(\xi_{1}+\frac{\lambda_{l}^{2}}{\lambda_{c}^{2}}\right)\left(\frac{\partial^{4}\tilde{w}\left(\tilde{x},t\right)}{\partial {\tilde{x}}^{2}\partial t^{2}}-{\tilde{g}}^{2}\;\frac{\partial^{6}\tilde{w}\left(\tilde{x},t\right)}{\partial {\tilde{x}}^{4}\partial t^{2}}\right) \end{array}$$

## 6. Higher-Order Hamiltonian Approach to Nonlinear Free Vibrations: Solution Procedure

Natural frequencies and mode shapes of flexural vibrations can be evaluated by employing the classical separation of the spatial and time variables

(28) $$\tilde{w}\left(\tilde{x},t\right)=W\left(\tilde{x}\right)\;e^{i\omega t}$$

being ω the natural frequency of flexural vibrations. Enforcing the separation of the variables Equation (28) to the differential condition of dynamic equilibrium, the governing equation of the linear flexural spatial mode shape for the NStressG model, $W\left(\tilde{x}\right)$, is obtained as

(29) $$\lambda_{c}^{2}\;\frac{\partial^{6}W\left(\tilde{x}\right)}{\partial {\tilde{x}}^{6}}+\frac{\partial^{4}W\left(\tilde{x}\right)}{\partial {\tilde{x}}^{4}}\left({\tilde{\omega }}^{2}\;\left(\lambda_{c}^{2}\;\xi_{1}+\lambda_{l}^{2}\right){\tilde{g}}^{2}+\left(\lambda_{c}^{2}\;\xi_{1}+\lambda_{l}^{2}\right)\left(\tilde{N^{T}}+\tilde{N^{C}}\right)-1\right)\;\;-\frac{\partial^{2}W\left(\tilde{x}\right)}{{\tilde{x}}^{2}}\left({\tilde{\omega }}^{2}\left(\lambda_{c}^{2}\;\xi_{1}+\lambda_{l}^{2}\right)+{\tilde{g}}^{2}{\tilde{\omega }}^{2}+\left(\tilde{N^{T}}+\tilde{N^{C}}\right)\right)+{\tilde{\omega }}^{2}W\left(\tilde{x}\right)=0$$

The analytical solution of the governing equation of the flexural spatial mode shape Equation (29) can be expressed by

(30) $$W\left(\tilde{x}\right)=\overset{6}{\underset{k=1}{\sum }}q_{k}e^{x\;\beta_{k}}\;$$

wherein $\beta_{k}\;$are the roots of the characteristic equation, and $q_{k}$ are six unknown constants to be determined by imposing the standard boundary conditions and the constitutive boundary conditions associated with NStressG.

Equation (26) describes the nonlinear free vibrations in the NStressG model of elasticity and in a hygrothermal environment. On the basis of the Galerkin method, the transverse displacement function $\tilde{w}\left(\tilde{x},t\right)$ in Equation (26) can be defined by

(31) $$\tilde{w}\left(\tilde{x},t\right)=\overset{N}{\underset{i=1}{\sum }}W_{i}\left(\tilde{x}\right)\;W_{i}\left(t\right)$$

where $W_{i}\left(\tilde{x}\right)$ is the i-*th* test function which depends on the assigned boundary conditions (Equation (30)) and $W_{i}\left(t\right)$ is the unknown i-*th* time-dependent coefficient.

In this study, we assume the test function form to be equal to the NStressG linear modal shape (*i =* 1)

(32) $$\tilde{w}\left(\tilde{x},t\right)=W_{1}\left(\tilde{x}\right)\;W_{1}\left(t\right)$$

### 6.1. First-Order Hamiltonian Approach 

Based on the First-order Hamiltonian approach introduced by [49], the time base function, $W_{1}\left(t\right),$ is given by the following approximate cosine solution

(33) $$W_{1}\left(t\right)=A_{w}cos\left(\omega_{1}t\right)$$

being $\omega_{1}$ the first nonlinear vibration frequency, $A_{w}\;$the amplitude of the nonlinear oscillator; moreover $W_{1}\left(\tilde{x}\right)$ is assumed to be equal to the linear spatial mode based on the NStressG model of elasticity

(34) $$W_{1}\left(\tilde{x}\right)=q_{1}e^{-\tilde{x}\beta_{1}}+q_{2}e^{\tilde{x}\beta_{1}}+q_{3}e^{-\tilde{x}\beta_{2}}+q_{4}e^{\tilde{x}\beta_{2}}+q_{5}e^{-\tilde{x}\beta_{3}}+q_{6}e^{\tilde{x}\beta_{3}}\;$$

Now, substituting Equation (32) into Equation (27) and multiplying the resulting equation with the fundamental vibration mode $W_{1}\left(\tilde{x}\right)$, then integrating across the length of the nanobeam, leads to the following equation

(35) $$\delta_{0}+\delta_{1}W_{1}\left(t\right)+\delta_{2}W_{1}^{2}\left(t\right)+\delta_{3}W_{1}^{3}\left(t\right)+W_{1}{}^{\prime \prime }\left(t\right)=0\;$$

where $\delta_{0},\delta_{1},\delta_{2},\;\mathrm{and}\;\delta_{3}$ are four coefficients obtained by splitting up the terms. 

Finally, in agreement with Hamiltonian approach to nonlinear oscillators [49], it is easy to establish a variational principle for Equation (35) [50]

(36) H=∫0T4δ0W1t+12δ1W12t+13δ2W1t+14δ3W1t−12W1′t2dt  

where T is the period of the nonlinear oscillator.

The frequency–amplitude relationship can be obtained from the following equation

(37) $$\frac{\partial }{\partial A_{w}}\left(\frac{\partial H}{\partial \frac{1}{\omega_{1}}}\right)=0\;$$

which gives the approximate nonlinear fundamental vibration frequency of a porous FG nano-beam

(38) $$\omega_{1}=\frac{\sqrt{-48\delta_{0}-12\pi A_{w}\delta_{1}-32A_{w}^{2}\delta_{2}-9\pi A_{w}^{3}\delta_{3}}}{2\sqrt{3\pi }\sqrt{A_{w}}}\;$$

Note that the linear vibration frequency of a porous FG nano-beam can be determined from the previous Equation (38) by setting $A_{w}=0$. 

### 6.2. Second-Order Hamiltonian Approach

In order to find the Second-order approximate solution and frequency, we assume that a Second-order trial solution can be expressed by

(39) $$W_{1}\left(t\right)=A_{1}cos\left(\omega_{1}t\right)+A_{2}cos\left(3\omega_{1}t\right)\;$$

with the following initial condition

(40) $$A_{w}=A_{1}+A_{2}\;$$

Applying the mathematical resolution method previously introduced for the First-order Hamiltonian approach [51], we obtain the following system of equations

(41) $$\left\{\begin{array}{c} \frac{\partial }{\partial A_{1}}\left(\frac{\partial H}{\partial \frac{1}{\omega_{1}}}\right)=0\\ \frac{\partial }{\partial A_{2}}\left(\frac{\partial H}{\partial \frac{1}{\omega_{1}}}\right)=0 \end{array}\right.\;$$

Solving Equations (40) and (41) simultaneously, and assuming Equation (39), one can obtain the Second-order solution and the approximate frequency $\omega_{1}$ according to the Hamiltonian approach.

### 6.3. Third-Order Hamiltonian Approach

The accuracy of the results will be further improved by consider the following equation as the response of the system

(42) $$W_{1}\left(t\right)=A_{1}cos\left(\omega_{1}t\right)+A_{2}cos\left(3\omega_{1}t\right)+A_{3}cos\left(5\omega_{1}t\right)\;$$

where the initial condition is

(43) $$A_{w}=A_{1}+A_{2}+A_{3}$$

By using the same procedure explained above (§ 6.2), the following system of equations follows

(44) $$\left\{\begin{array}{c} \frac{\partial }{\partial A_{1}}\left(\frac{\partial H}{\partial \frac{1}{\omega_{1}}}\right)=0\\ \frac{\partial }{\partial A_{2}}\left(\frac{\partial H}{\partial \frac{1}{\omega_{1}}}\right)=0\\ \frac{\partial }{\partial A_{3}}\left(\frac{\partial H}{\partial \frac{1}{\omega_{1}}}\right)=0 \end{array}\right.\;$$

Similarly, by solving Equation (44) simultaneously with Equation (43), the amplitude-frequency relation up to the Third-order approximation is obtained.

## 7. Convergence and Comparison Study

In order to validate the accuracy and reliability of the proposed approach, three numerical examples are presented in this paragraph.

To this purpose, both a uniform temperature rise, $T\left(z^{\prime }\right)=T_{b}+\Delta T,\;$and a moisture concentration, $C\left(z^{\prime }\right)=C_{b}+\Delta C,\;$between the bottom (*z’* = −h/2) and the top surface (z’ = +h/2) of the nano-beam cross-section, are considered (Figure 1), $T_{b}=305\;\left[K\right]$ and $C_{b}=0\;\left[\mathrm{wt}.{\%H}_{2}O\right]$ being the reference values of the temperature and moisture concentration at the bottom surface, respectively, and ΔT, ΔC their increments.

In the first two comparison examples, the normalized frequency ratio between the dimensionless nonlocal fundamental frequency, $\tilde{\omega }$, and the dimensionless local natural frequency,$\;{\tilde{\omega }}_{loc}$, of a clamped–clamped (C–C) porous FG nano-beam in a hygrothermal environment, were compared (Table 3 and Table 4), with the results obtained by Penna et al. in [46] for *λ_c_* = 0.2 and assuming: *λ_l_* = 0.0 or 0.10; ξ_1_ = 0.0 or 0.5; Δ*T* = 0, 50, and 100 [K].

In the third example (Table 5), the present approach is compared with the model proposed by Barretta et al. in [42] for a C–C porous FG nano-beam in absence of hygrothermal loads for *λ_l_* = 0.1, varying *λ_c_,* in the set {0.0^+^, 0.2, 0.4, 0.6, 0.8, 1.0} and assuming ξ_1_ = 0.0 or 0.5, and the gyration radius, $\tilde{g}$, equal to 1/20. 

Moreover, Table 6, Table 7 and Table 8 summarize the linear frequency values assuming $A_{w}=0$.

From these comparison examples, the accuracy of the higher order Hamiltonian approach to the nonlinear oscillators here employed is validated.

## 8. Results and Discussion

The effects of the hygrothermal loads on the nonlinear dynamic behavior of a C–C Bernoulli–Euler porous FG nano-beam is discussed here, varying the nonlocal parameter, *λ_c_*, the gradient length parameter, *λ_l_*, the mixture parameter, *ξ*_1_, and the nonlinear oscillator amplitude, $A_{w\;}$.

In particular, the dimensionless nonlocal fundamental frequency has been evaluated assuming *k* = 0.3 and *ζ* = 0.15 with a temperature increment ΔT ranging in the set {0, 50, 100 [K]} and considering *C =* 2 [wt.%H_2_O]. Moreover, we have also investigated the effects of the porosity volume fraction, *ζ*, the gradient index, *k*, and temperature rise on the dimensionless bending stiffness, $\left(\bar{I_{E}}=\frac{I_{E}}{I_{E}{}_{c}}\right)$, the dimensionless axial stiffness, $\left(\bar{A_{E}}=\frac{A_{E}}{A_{E_{c}}}\right)$, the dimensionless effective cross sectional mass, $\left(\bar{A_{\rho }}=\frac{A_{\rho }}{A_{\rho_{c}}}\right)$, and the dimensionless rotary inertia, $\left(\bar{I_{\rho }}=\frac{I_{\rho }}{I_{\rho_{c}}}\right)$. Note that $I_{E}{}_{c}\;$and $A_{E_{c}}\;$represent the bending and axial stiffness of a non-porous purely ceramic nano-beam, respectively, while $A_{\rho_{c}}$, $I_{\rho_{c}}$ are the effective cross-sectional mass and rotary inertia of a non-porous purely ceramic nano-beam, respectively.

### 8.1. Influence of Porosity Volume Fraction and Gradient Index

**Figure 2:**
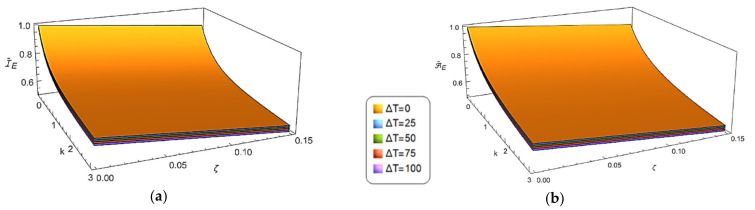
Combined effects of the gradient index (*k*) and the porosity volume fraction (ζ) on the dimensionless bending stiffness $\bar{I_{E}}$ (**a**) and axial stiffness $\bar{A_{E}}$ (**b**) under uniform temperature rises (ΔT = 0, 25, 50, 75, 100 [K]).

**Figure 3:**
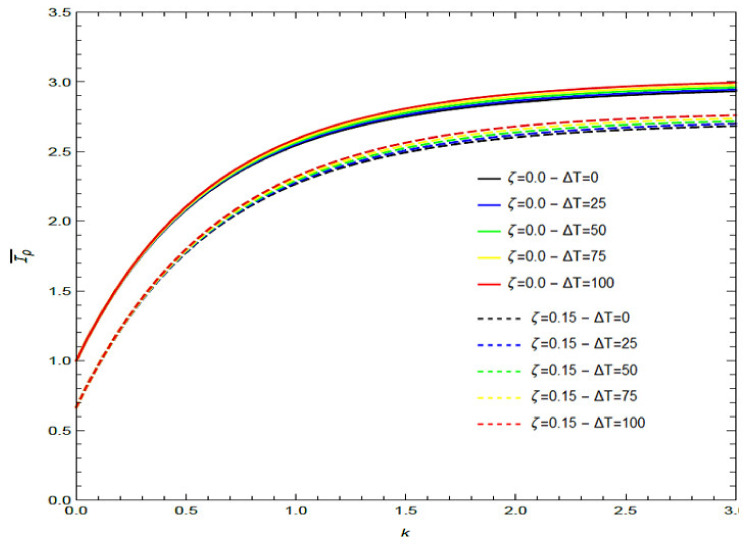
Combined effects of the gradient index (*k*) and the porosity volume fraction (ζ) on the dimensionless rotary inertia $\bar{I_{\rho }}$ under uniform temperature rises (ΔT = 0, 25, 50, 75, 100 [K]).

**Figure 4:**
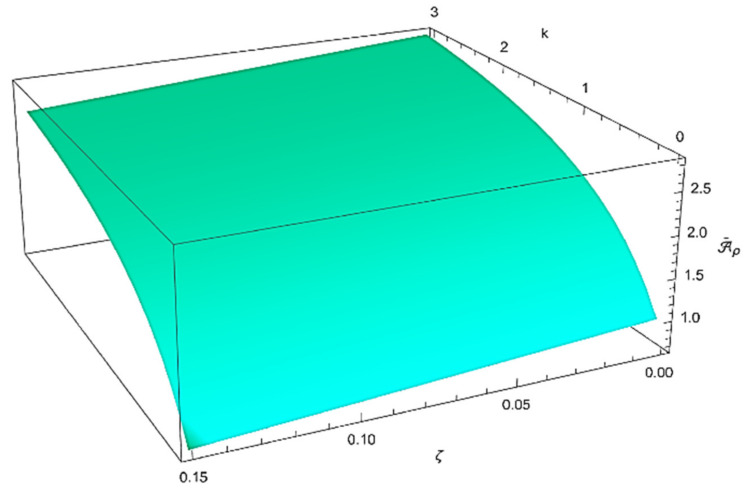
Combined effect of the gradient index (*k*) and the porosity volume fraction (ζ) on the dimensionless effective cross-sectional mass $\bar{A_{\rho }}$.

The combined effects of both the gradient index, k, and the porosity volume fraction, ζ, on the thermo-mechanical properties of the porous FG nanobeam under investigation are presented in Figure 2, Figure 3 and Figure 4. It can be noted how the dimensionless bending and axial stiffnesses, as well as the dimensionless rotary inertia and effective cross-sectional mass, decrease as the porosity volume fraction increases, while they increase as the material gradient index increases.

### 8.2. Influence of Hygrothermal Loads

In this subsection, the influence of hygrothermal loads on the normalized fundamental flexural frequency is discussed. Firstly, as can be observed from Table 9, Table 10, Table 11, Table 12, Table 13, Table 14, Table 15, Table 16 and Table 17, the values of the normalized linear fundamental flexural frequency ($A_{w\;}=0)$, based on a local/nonlocal stress-driven gradient theory of elasticity, always decrease as the temperature rise increases. Moreover, in the range of values here considered, an opposite trend is obtained for the normalized nonlinear fundamental flexural frequency as $A_{w\;}$and ΔT increase. 

With reference to the influence of the temperature on the thermo-mechanical properties of the porous FG nanobeam, it can be observed (Figure 2) that the dimensionless bending stiffness and dimensionless axial stiffness decrease as ΔT increases. In addition, the curves of Figure 3 show that the dimensionless rotary inertia increases as the temperature increases, although the hygrothermal effect is noticeable when *k* > 1.

### 8.3. Influence of Nonlocal Parameter, Gradient Length Parameter, and Mixture Parameter

From Table 9, Table 10, Table 11, Table 12, Table 13, Table 14, Table 15, Table 16 and Table 17, on one hand, it can be seen that an increase in the values of $\lambda_{c}$ results in an increase of the frequency ratio, $\tilde{\omega }$/${\tilde{\omega }}_{loc}$, but on the other, it can be found that as $\lambda_{l}\;$increases, the values of the aforementioned frequency ratio decrease. It is also possible to note that the ratio $\tilde{\omega }$/${\tilde{\omega }}_{loc}$, decreases by increasing the mixture parameter *ξ_1_*.

### 8.4. Influence of Higher-Order Hamilton Approach

Finally, the nonlinear dimensionless natural frequencies of the porous FG nano-beam under investigation corresponding to the First-, Second-, and Third-order approximate solutions are summarized in Table 9, Table 10, Table 11, Table 12, Table 13, Table 14, Table 15, Table 16 and Table 17, varying the oscillator amplitude in the set {0.0, 0.01, 0.05, 0.10}. From these tables, it can be seen that the aforementioned flexural frequency always increase as the amplitude of the nonlinear oscillator increases, while they decrease as the order of the Hamiltonian approach increases. 

The above parametrical analysis assumes relevance in the study of the nonlinear vibrations of porous FG nano-beams because their behavior is influenced by the dimensionless term ${\tilde{r}}^{2}$, which is proportional to the ratio between the axial and the bending stiffness of the nanobeam cross-section, both depending on the porosity distribution of the structure of the nano-beam material and on the temperature increment and the material gradient index. Moreover, the term ${\tilde{r}}^{2}$ allows us to take into account the nonlinear response due to the mid-plane stretching effect introduced in the following Appendix A.

## 9. Conclusions

In this paper, the nonlinear dynamic behavior of a Bernoulli–Euler nano-beam made of a metal–ceramic functionally graded porous material in a hygrothermal environment, with von Kármán type nonlinearity, was studied, employing the local/nonlocal stress-driven integral model.

The governing equations have been reduced to a nonlinear ordinary differential equation by using the Galerkin method. Then, the higher-order Hamiltonian approach to nonlinear oscillators was employed.

In view of the numerical results obtained in the present study, the following main conclusions may be formulated:

(1) the flexural frequency always increases with the increase of the nonlocal parameter;
(2) the flexural frequency decreases always by increasing the gradient length parameter;
(3) an increase in the values of the mixture parameter always leads to a decrease in the flexural frequency;
(4) the flexural frequency always increases as the amplitude of the nonlinear oscillator increases, while they decrease as the order of the Hamiltonian approach increases.

In conclusion, the results obtained in this study show that the proposed approach is capable of capturing the nonlinear dynamic behavior of porous Bernoulli–Euler functionally graded nano-beams in a hygrothermal environment and represent a valuable reference point for engineers and researchers to validate different numerical methods, as well as for the practical design of nano-scaled beam-like components of nano electromechanical systems (NEMS).

## Figures and Tables

**Table 1:** Characteristic values of thermo-elastic properties ($f_{c},\;f_{m}$ ) of ceramic (Si_3_N_4_) and metal (SuS_3_O_4_) [46].

Material	$\mathrm{Properties}\;(f_{c},\;f_{m})$	Unit	$P_{0}$
Ceramic (Si_3_N_4_)	E_c_	[GPa]	348.40
	ρ_c_	[kg/m^3^]	2325
	α_c_	[K^−1^]	5.87 × 10^−6^
	β_c_	[wt.% H_2_O]^−1^	0
Metal (SuS_3_O_4_)	E_m_	[GPa]	201.04
	ρ_m_	[kg/m^3^]	8011
	α_m_	[K^−1^]	1.233 × 10^−5^
	β_m_	[wt.% H_2_O]^−1^	5 × 10^−4^

**Table 2:** Coefficients of material phases ($X_{-1},X_{1},X_{2}$, $X_{3}$) for ceramic (Si_3_N_4_) and metal (SuS_3_O_4_).

		Ceramic (Si_3_N_4_)				Metal (SuS_3_O_4_)			
Coefficients	Unit	E_c_	ρ_c_	α_c_	β_c_	E_m_	ρ_m_	α_m_	β_m_
X_−1_	[K]	0	0	0	0	0	0	0	0
X_1_	[K^−1^]	−3.07 × 10^−4^	0	9.095 × 10^−4^	0	3.079 × 10^−4^	0	8.086 × 10^−4^	0
X_2_	[K^−2^]	2.160 × 10^−7^	0	0	0	−6.534 × 10^−7^	0	0	0
X_3_	[K^−3^]	−8.946 × 10^−11^	0	0	0	0	0	0	0

**Table 3:** Linear dimensionless natural frequencies of porous FG clamped–clamped (C–C) nano-beam ($A_{w}=0,\;\lambda_{c}=0.20,\;\xi_{1}=0.0$ ).

$\lambda_{l}$	ξ_1_ = 0.0					
	Present Approach	Ref. [46]	Present Approach	Ref. [46]	Present Approach	Ref. [46]
	ΔT=0		ΔT=50		ΔT=100	
0.00	1.83226	1.83226	1.82706	1.82706	1.82313	1.82313
0.10	1.57333	1.57333	1.56718	1.56718	1.56254	1.56254

**Table 4:** Linear dimensionless natural frequencies of porous FG clamped–clamped (C–C) nano-beam ($A_{w}=0,\;\lambda_{c}=0.20,\;\xi_{1}=0.5$ ).

$\lambda_{l}$	ξ_1_ = 0.5					
	Present Approach	Ref. [46]	Present Approach	Ref. [46]	Present Approach	Ref. [46]
	ΔT=0		ΔT=50		ΔT=100	
0.00	1.23148	1.23148	1.22424	1.22424	1.21876	1.21876
0.10	1.13883	1.13883	1.13089	1.13089	1.12487	1.12487

**Table 5:** Linear dimensionless natural frequencies of porous FG clamped–clamped (C–C) nano-beam ($A_{w}=\Delta T=0,\;\tilde{g}=\frac{1}{20},\;\lambda_{l}=0.10$ ).

λ_c_	ξ_1_ = 0.0		ξ_1_ = 0.5	
	Present Approach	Ref. [42]	Present Approach	Ref. [42]
0.0^+^	0.89165	0.89165	0.88416	0.88416
0.20	1.58127	1.58127	1.14531	1.14531
0.40	2.57577	2.57577	1.28946	1.28946
0.60	3.61940	3.61940	1.34633	1.34633
0.80	4.67784	4.67784	1.37237	1.37237
1.00	5.74258	5.74258	1.38608	1.38608

**Table 6:** Linear dimensionless natural frequencies of porous FG clamped–clamped (C–C) nano-beam for ξ_1_ = 0.0.

$A_{w}=0$	ξ_1_ = 0.0					
λ_c_	ΔT=0		ΔT=50		ΔT=100	
	$\lambda_{l}=0.00$	$\lambda_{l}=0.10$	$\lambda_{l}=0.00$	$\lambda_{l}=0.10$	$\lambda_{l}=0.00$	$\lambda_{l}=0.10$
0.10	1.33333	1.15406	1.32613	1.14551	1.32070	1.13904
0.20	1.84414	1.58369	1.83894	1.57754	1.83504	1.57291

**Table 7:** Linear dimensionless natural frequencies of porous FG clamped–clamped (C–C) nano-beam for ξ_1_ = 0.5.

$A_{w}=0$	ξ_1_ = 0.5					
λ_c_	ΔT=0		ΔT=50		ΔT=100	
	$\lambda_{l}=0.00$	$\lambda_{l}=0.10$	$\lambda_{l}=0.00$	$\lambda_{l}=0.10$	$\lambda_{l}=0.00$	$\lambda_{l}=0.10$
0.10	1.12891	1.01093	1.12085	1.00166	1.11477	0.99464
0.20	1.23896	1.14585	1.23170	1.13789	1.22623	1.13187

**Table 8:** Linear dimensionless natural frequencies of porous FG clamped–clamped (C–C) nano-beam for ξ_1_ = 1.0.

$A_{w}=0$	ξ_1_ = 1.0					
λ_c_	ΔT=0		ΔT=50		ΔT=100	
	$\lambda_{l}=0.00$	$\lambda_{l}=0.10$	$\lambda_{l}=0.00$	$\lambda_{l}=0.10$	$\lambda_{l}=0.00$	$\lambda_{l}=0.10$
0.10	0.99999	0.91331	0.99115	0.90336	0.98444	0.89581
0.20	1.11740	0.94718	1.13331	0.93774	1.14511	0.93058

**Table 9:** Nonlinear dimensionless natural frequencies of porous FG clamped–clamped (C–C) nano-beam for ξ_1_ = 0.0 in the case of First-Order Hamiltonian Approach.

ξ_1_ = 0.0	$A_{w}$	ΔT=0		ΔT=50		ΔT=100	
		$\lambda_{l}=0.00$	$\lambda_{l}=0.10$	$\lambda_{l}=0.00$	$\lambda_{l}=0.10$	$\lambda_{l}=0.00$	$\lambda_{l}=0.10$
λ_c_ = 0.1	0.00	1.33333	1.15406	1.32613	1.14551	1.32070	1.13904
	0.01	1.33469	1.15575	1.32761	1.14769	1.32236	1.14117
	0.05	1.36706	1.19553	1.36270	1.19886	1.36164	1.19121
	0.10	1.46359	1.31211	1.46697	1.34630	1.47766	1.33554
λ_c_ = 0.2	0.00	1.84414	1.58369	1.83894	1.57754	1.83504	1.57291
	0.01	1.84464	1.58430	1.83950	1.57821	1.83564	1.57364
	0.05	1.85680	1.59886	1.85269	1.59406	1.85004	1.59117
	0.10	1.89429	1.64355	1.89333	1.64262	1.89435	1.64471

**Table 10:** Nonlinear dimensionless natural frequencies of porous FG clamped–clamped (C–C) nano-beam for ξ_1_ = 0.5 in the case of First-Order Hamiltonian Approach.

ξ_1_ = 0.5	$A_{w}$	ΔT=0		ΔT=50		ΔT=100	
		$\lambda_{l}=0.00$	$\lambda_{l}=0.10$	$\lambda_{l\;}=0.00$	$\lambda_{l}=0.10$	$\lambda_{l}=0.00$	$\lambda_{l}=0.10$
λ_c_ = 0.1	0.00	1.12891	1.01093	1.12085	1.00166	1.11477	0.99464
	0.01	1.13040	1.01267	1.12250	1.00362	1.11666	0.99695
	0.05	1.16559	1.05373	1.16131	1.04952	1.16128	1.05087
	0.10	1.26930	1.17279	1.27499	1.18153	1.29082	1.20390
λ_c_ = 0.2	0.00	1.23896	1.14585	1.23170	1.13789	1.22623	1.13187
	0.01	1.23965	1.14660	1.23247	1.13872	1.22711	1.13284
	0.05	1.25622	1.16447	1.25082	1.15865	1.24806	1.15590
	0.10	1.30663	1.21862	1.30650	1.21884	1.31137	1.22516

**Table 11:** Nonlinear dimensionless natural frequencies of porous FG clamped–clamped (C–C) nano-beam for ξ_1_ = 1.0 in the case of First-Order Hamiltonian Approach.

ξ_1_ = 1.0	$A_{w}$	ΔT=0		ΔT=50		ΔT=100	
		$\lambda_{l}=0.00$	$\lambda_{l}=0.10$	$\lambda_{l}=0.00$	$\lambda_{l}=0.10$	$\lambda_{l}=0.00$	$\lambda_{l}=0.10$
λ_c_ = 0.1	0.00	0.99999	0.91331	0.99115	0.90336	0.98444	0.89581
	0.01	1.00161	0.91514	0.99296	0.90544	0.98658	0.89832
	0.05	1.03951	0.95786	1.03544	0.95401	1.03662	0.95670
	0.10	1.14994	1.08052	1.15820	1.09195	1.17937	1.11968
λ_c_ = 0.2	0.00	1.11740	0.94718	1.13331	0.93774	1.14511	0.93058
	0.01	1.11837	0.94804	1.13444	0.93872	1.14645	0.93176
	0.05	1.14139	0.96837	1.16110	0.96200	1.17811	0.95983
	0.10	1.21050	1.02932	1.24073	1.03137	1.27198	1.04266

**Table 12:** Nonlinear dimensionless natural frequencies of porous FG clamped–clamped (C–C) nano-beam for ξ_1_ = 0.0 in the case of Second-Order Hamiltonian Approach.

ξ_1_ = 0.0	$A_{w}$	ΔT=0		ΔT=50		ΔT=100	
		$\lambda_{l}=0.00$	$\lambda_{l}=0.10$	$\lambda_{l}=0.00$	$\lambda_{l}=0.10$	$\lambda_{l}=0.00$	$\lambda_{l}=0.10$
λ_c_ = 0.1	0.00	1.33333	1.15406	1.32613	1.14551	1.32070	1.13904
	0.01	1.33469	1.15575	1.32761	1.14769	1.32236	1.41117
	0.05	1.36699	1.19542	1.36263	1.19868	1.36154	1.19103
	0.10	1.46272	1.31073	1.46596	1.34421	1.47644	1.33352
λ_c_ = 0.2	0.00	1.84414	1.58369	1.83894	1.57754	1.83504	1.57291
	0.01	1.84464	1.58430	1.83950	1.57821	1.83564	1.57364
	0.05	1.85679	1.59885	1.85268	1.59405	1.85003	1.59115
	0.10	1.89418	1.64338	1.89320	1.64241	1.89421	1.64447

**Table 13:** Nonlinear dimensionless natural frequencies of porous FG clamped–clamped (C–C) nano-beam for ξ_1_ = 0.5 in the case of Second-Order Hamiltonian Approach.

ξ_1_ = 0.5	$A_{w}$	ΔT=0		ΔT=50		ΔT=100	
		$\lambda_{l}=0.00$	$\lambda_{l}=0.10$	$\lambda_{l}=0.00$	$\lambda_{l}=0.10$	$\lambda_{l}=0.00$	$\lambda_{l}=0.10$
λ_c_ = 0.1	0.00	1.12891	1.01093	1.12085	1.00166	1.11477	0.99464
	0.01	1.13040	1.01267	1.12250	1.00362	1.11666	0.99695
	0.05	1.16550	1.05359	1.16199	1.04935	1.16133	1.05064
	0.10	1.26817	1.17121	1.27364	1.17962	1.28911	1.20143
λ_c_ = 0.2	0.00	1.23896	1.14585	1.23170	1.13789	1.22623	1.13187
	0.01	1.23965	1.14660	1.23247	1.13872	1.22711	1.13284
	0.05	1.25620	1.16445	1.25080	1.15862	1.24802	1.15585
	0.10	1.30635	1.21828	1.30616	1.21842	1.31904	1.22461

**Table 14:** Nonlinear dimensionless natural frequencies of porous FG clamped–clamped (C–C) nano-beam for ξ_1_ = 1.0 in the case of Second-Order Hamiltonian Approach.

ξ_1_ = 1.0	$A_{w}$	ΔT=0		ΔT=50		ΔT=100	
		$\lambda_{l}=0.00$	$\lambda_{l}=0.10$	$\lambda_{l}=0.00$	$\lambda_{l}=0.10$	$\lambda_{l}=0.00$	$\lambda_{l}=0.10$
λ_c_ = 0.1	0.00	0.99999	0.91331	0.99115	0.90336	0.98444	0.89581
	0.01	1.00161	0.91514	0.99296	0.90544	0.98658	0.89832
	0.05	1.03939	0.95769	1.03529	0.95380	1.03641	0.95640
	0.10	1.14854	1.07872	1.15650	1.08974	1.17716	1.11674
λ_c_ = 0.2	0.00	1.11740	0.94718	1.13331	0.93774	1.14511	0.93058
	0.01	1.11837	0.94804	1.13444	0.93872	1.14645	0.93176
	0.05	1.14135	0.96833	1.16105	0.96195	1.17804	0.95975
	0.10	1.20955	1.02882	1.24003	1.03073	1.27104	1.04178

**Table 15:** Nonlinear dimensionless natural frequencies of porous FG clamped–clamped (C–C) nano-beam for ξ_1_ = 0.0 in the case of Third-Order Hamiltonian Approach.

ξ_1_ = 0.0	$A_{w}$	ΔT=0		ΔT=50		ΔT=100	
		$\lambda_{l\;}=0.00$	$\lambda_{l}=0.10$	$\lambda_{l}=0.00$	$\lambda_{l}=0.10$	$\lambda_{l\;}=0.00$	$\lambda_{l}=0.10$
λ_c_ = 0.1	0.00	1.33333	1.15406	1.32613	1.14551	1.32070	1.13904
	0.01	1.33469	1.15575	1.32761	1.14769	1.32236	1.14117
	0.05	1.36699	1.19542	1.36262	1.19867	1.36154	1.19102
	0.10	1.46271	1.31070	1.46595	1.34416	1.47642	1.33347
λ_c_ = 0.2	0.00	1.84414	1.58369	1.83894	1.57754	1.83504	1.57291
	0.01	1.84464	1.58430	1.83850	1.57821	1.83564	1.57364
	0.05	1.85679	1.59885	1.85268	1.59405	1.85003	1.59115
	0.10	1.89417	1.64337	1.89319	1.64241	1.89420	1.64446

**Table 16:** Nonlinear dimensionless natural frequencies of porous FG clamped–clamped (C–C) nano-beam for ξ_1_ = 0.5 in the case of Third-Order Hamiltonian Approach.

ξ_1_ = 0.5	$A_{w}$	ΔT=0		ΔT=50		ΔT=100	
		$\lambda_{l}=0.00$	$\lambda_{l}=0.10$	$\lambda_{l}=0.00$	$\lambda_{l}=0.10$	$\lambda_{l\;}=0.00$	$\lambda_{l}=0.10$
λ_c_ = 0.1	0.00	1.12891	1.01093	1.12085	1.00166	1.11477	0.99464
	0.01	1.13040	1.01267	1.12250	1.00362	1.11666	0.99695
	0.05	1.16550	1.05359	1.16199	1.04935	1.16113	1.05087
	0.10	1.26815	1.17117	1.27362	1.17958	1.28907	1.20390
λ_c_ = 0.2	0.00	1.23896	1.14585	1.23170	1.13789	1.22623	1.13187
	0.01	1.23965	1.14660	1.23247	1.13872	1.22711	1.13284
	0.05	1.25620	1.16445	1.25080	1.15862	1.24802	1.15585
	0.10	1.30634	1.21827	1.30615	1.21841	1.31093	1.22460

**Table 17:** Nonlinear dimensionless natural frequencies of porous FG clamped–clamped (C–C) nano-beam for ξ_1_ = 1.0 in the case of Third-Order Hamiltonian Approach.

ξ_1_ = 1.0	$A_{w}$	ΔT=0		ΔT=50		ΔT=100	
		$\lambda_{l\;}=0.00$	$\lambda_{l}=0.10$	$\lambda_{l}=0.00$	$\lambda_{l}=0.10$	$\lambda_{l}=0.00$	$\lambda_{l}=0.10$
λ_c_ = 0.1	0.00	0.99999	0.91331	0.99115	0.90336	0.98444	0.89581
	0.01	1.00161	0.91514	0.99296	0.90544	0.98658	0.89832
	0.05	1.03939	0.95769	1.03529	0.95380	1.03641	0.95640
	0.10	1.14851	1.07868	1.15646	1.08968	1.17711	1.11665
λ_c_ = 0.2	0.00	1.11740	0.94718	1.13331	0.93774	1.14511	0.93058
	0.01	1.11837	0.94804	1.13444	0.93872	1.14645	0.97176
	0.05	1.14135	0.96833	1.16105	0.96195	1.17804	0.95975
	0.10	1.20954	1.02881	1.24002	1.03072	1.27103	1.04176

## Data Availability

The data presented in this study are available on request from the corresponding author.

## Appendix A

In this appendix, we report the mathematical steps taken to arrive at the equation that governs the problem of nonlinear transverse free vibrations of the nano-beam studied.

By manipulating Equation (6) and substituting into Equations (19)–(21), then multiplying by (1, z), the integration over the nano-beam cross section provides the following NStressG equations in terms of axial and transverse displacement

(A1) $$\varepsilon^{\left(vK\right)}\left(x,t\right)-L_{c}^{2}\;\frac{\partial^{2}\;\varepsilon^{\left(vK\right)}\left(x,t\right)}{\partial x^{2}}=\frac{N^{NStressG}\left(x,t\right)}{A_{E}}-\frac{L_{c}^{2}}{A_{E}}\left(\xi_{1}+\frac{L_{l}^{2}}{L_{c}^{2}}\right)\frac{\;\partial^{2}N^{NStressG}\left(x,t\right)}{\partial x^{2}}$$

(A2) $$-\chi \left(x,t\right)+L_{c}^{2}\;\frac{\partial^{2}\chi \left(x,t\right)}{\partial x^{2}}=\frac{M^{NStressG}\left(x,t\right)}{I_{E}}-\frac{L_{c}^{2}}{I_{E}}\left(\xi_{1}+\frac{L_{l}^{2}}{L_{c}^{2}}\right)\frac{\partial^{2}M^{NStressG}\left(x,t\right)}{\partial x^{2}}$$

with the following constitutive boundary conditions (CBC)

(A3) $$\frac{\partial \varepsilon^{\left(vK\right)}\;}{\partial x}\left(0,t\right)-\frac{1}{L_{c}}\varepsilon^{\left(vK\right)}\left(0,t\right)=-\frac{1}{A_{E}}\frac{\xi_{1}}{L_{c}}N^{NStressG}\left(0,t\right)+\frac{1}{A_{E}}\left(\xi_{1}+\frac{L_{l}^{2}}{L_{c}^{2}}\right)\;\frac{\partial N^{NStressG}\left(0,t\right)}{\partial x}$$

(A4) $$\frac{\partial \varepsilon^{\left(vK\right)}\;}{\partial x}\left(L,t\right)+\frac{1}{L_{c}}\varepsilon^{\left(vK\right)}\left(L,t\right)=\frac{1}{A_{E}}\frac{\xi_{1}}{L_{c}}N^{NStressG}\left(L,t\right)+\frac{1}{A_{E}}\;\left(\xi_{1}+\frac{L_{l}^{2}}{L_{c}^{2}}\right)\;\frac{\partial N^{NStressG}\left(L,t\right)}{\partial x}$$

(A5) $$-\frac{\partial \chi \;}{\partial x}\left(0,t\right)+\frac{1}{L_{c}}\chi \left(0,t\right)=-\frac{1}{I_{E}}\frac{\xi_{1}}{L_{c}}M^{NStressG}\left(0,t\right)+\frac{1}{I_{E}}\;\left(\xi_{1}+\frac{L_{l}^{2}}{L_{c}^{2}}\right)\;\frac{\partial M^{NStressG}\left(0,t\right)}{\partial x}$$

(A6) $$-\frac{\partial \chi \;}{\partial x}\left(L,t\right)-\frac{1}{L_{c}}\chi \left(L,t\right)=\frac{1}{I_{E}}\frac{\xi_{1}}{L_{c}}M^{NStressG}\left(L,t\right)+\frac{1}{I_{E}}\left(\xi_{1}+\frac{L_{l}^{2}}{L_{c}^{2}}\right)\;\frac{\partial M^{NStressG}\left(L,t\right)}{\partial x}$$

By manipulating the nonlinear equations of motion (Equations (9) and (10)), as well as Equations (A1) and (A2), we obtain the expression of nonlocal axial force and moment resultant in the NStressG model of elasticity

(A7) $$N^{NstressG}\left(x,t\right)=L_{c}^{2}\left(\xi_{1}+\frac{L_{l}^{2}}{L_{c}^{2}}\right)A_{\rho }\frac{\partial^{3}u}{\partial x\partial t^{2}}+A_{E}\left(\varepsilon^{\left(vK\right)}-L_{c}^{2}\frac{\partial^{2}}{\partial x^{2}}\;\varepsilon^{\left(vK\right)}\right)$$

(A8) $$M^{NstressG}\left(x,t\right)=-I_{E}\;\chi +{\;I}_{E}L_{c}^{2}\;\frac{\partial^{2}\chi \;}{\partial x^{2}}+L_{c}^{2}\left(\xi_{1}+\frac{L_{l}^{2}}{L_{c}^{2}}\right)\left(A_{\rho }\frac{\partial^{2}w}{\partial t^{2}}-I_{\rho }\;\frac{\partial^{4}w}{\partial x^{2}\partial t^{2}}-\frac{\partial }{\partial x}\left(N^{NstressG}\frac{\partial w}{\partial x}\right)+\left(N^{T}+N^{C}\right)\frac{\partial^{2}w}{\partial x^{2}}\right)$$

Moreover, by substituting Equations (A7) and (A8) into Equations (9) and (10), the following stress gradient equations of motion can be derived

(A9) $$A_{E}\left(\frac{\partial \varepsilon^{vK}}{\partial x}-L_{c}^{2}\;\frac{\partial^{3}\varepsilon^{vK}}{\partial x^{3}}\right)=A_{\rho }\frac{\partial^{2}u}{\partial t^{2}}-L_{c}^{2}\left(\xi_{1}+\frac{L_{l}^{2}}{L_{c}^{2}}\right)A_{\rho }\frac{\partial^{4}u}{\partial x^{2}\partial t^{2}}$$

(A10) $$\frac{\partial^{2}}{\partial x^{2}}\left(-I_{E}\;\chi +\;I_{E}L_{c}^{2}\;\frac{\partial^{2}\chi \;}{\partial x^{2}}+L_{c}^{2}\left(\xi_{1}+\frac{L_{l}^{2}}{L_{c}^{2}}\right)\left(A_{\rho }\frac{\partial^{2}w}{\partial t^{2}}-I_{\rho }\;\frac{\partial^{4}w}{\partial x^{2}\partial t^{2}}-\frac{\partial }{\partial x}\left(N^{NstressG}\frac{\partial w}{\partial x}\right)+\left(N^{T}+N^{C}\right)\frac{\partial^{2}w}{\partial x^{2}}\right)\right)\;\;=\;A_{\rho }\frac{\partial^{2}w}{\partial t^{2}}-I_{\rho }\;\frac{\partial^{4}w}{\partial x^{2}\;\partial t^{2}}-\frac{\partial }{\partial x}\left(N^{NstressG}\frac{\partial w}{\partial x}\right)+\left(N^{T}+N^{C}\right)\frac{\partial^{2}w}{\partial x^{2}}$$

Employing the axial and flexural kinematic compatibility, the differential condition of dynamic equilibrium governing the vibrations of NStressG nano-beams is given by

(A11) $$A_{E}\left(\frac{\partial^{2}u}{\partial x^{2}}+\frac{\partial }{\partial x}\left(\frac{1}{2}{\left(\frac{\partial w}{\partial x}\right)}^{2}\right)\;\right)-L_{c}^{2}A_{E}\left(\frac{\partial^{4}u}{\partial x^{4}}+\frac{\partial^{3}}{\partial x^{3}}\left(\frac{1}{2}{\left(\frac{\partial w}{\partial x}\right)}^{2}\right)\right)=A_{\rho }\frac{\partial^{2}u}{\partial t^{2}}-L_{c}^{2}\left(\xi_{1}+\frac{L_{l}^{2}}{L_{c}^{2}}\right)A_{\rho }\frac{\partial^{2}}{\partial x^{2}}\frac{\partial^{2}u}{\partial t^{2}}$$

(A12) $$-I_{E}\;\frac{\partial^{4}w\left(x,t\right)}{\partial x^{4}}+I_{E}L_{c}^{2}\frac{\partial^{6}w\left(x,t\right)}{\partial x^{6}}+L_{c}^{2}\left(\xi_{1}+\frac{L_{l}^{2}}{L_{c}^{2}}\right)\frac{\partial^{2}}{\partial x^{2}}\left(A_{\rho }\frac{\partial^{2}w}{\partial t^{2}}-I_{\rho }\;\frac{\partial^{4}w}{\partial x^{2}\;\partial t^{2}}-\frac{\partial }{\partial x}\left(N^{NstressG}\frac{\partial w}{\partial x}\right)+\left(N^{T}+N^{C}\right)\frac{\partial^{2}w}{\partial x^{2}}\right)\;=\left(A_{\rho }\frac{\partial^{2}w}{\partial t^{2}}-I_{\rho }\;\frac{\partial^{4}w}{\partial x^{2}\;\partial t^{2}}-\frac{\partial }{\partial x}\left(N^{NstressG}\frac{\partial w}{\partial x}\right)+\left(N^{T}+N^{C}\right)\frac{\partial^{2}w}{\partial x^{2}}\right)$$

with the following natural boundary conditions at the nano-beam ends $\left(x=0,L\right)$

(A13) $$N^{NstressG}\left(x,t\right)=\bar{N}$$

(A14) $$I_{\rho }\frac{\partial^{3}w\left(x,t\right)}{\partial x\;\partial t^{2}}-\left(N^{T}+N^{C}\right)\frac{\partial w}{\partial x}+\frac{\partial M^{NstressG}\left(x,t\right)}{\partial x}=\bar{V}$$

(A15) $$M^{NstressG}\left(x,t\right)=\bar{M}$$

being $\bar{N}$, $\bar{M}$ and$\;\bar{V}$ the assigned generalized forces acting at the nano-beam ends together and with the constitutive boundary conditions at the nano-beam ends given by Equations (A3)–(A6) which can be rewritten as a function of the displacement components

(A16) $$\frac{\partial }{\partial x}\;\left(\frac{\partial u\left(0,t\right)}{\partial x}+\frac{1}{2}{\left(\frac{\partial w\left(0,t\right)}{\partial x}\right)}^{2}\right)-\frac{1}{L_{c}}\left(\frac{\partial u\left(0,t\right)}{\partial x}+\frac{1}{2}{\left(\frac{\partial w\left(0,t\right)}{\partial x}\right)}^{2}\right)=-\frac{1}{A_{E}}\frac{\xi_{1}}{L_{c}}N^{NstressG}\left(0,t\right)+\frac{1}{A_{E}}\left(\xi_{1}+\frac{L_{l}^{2}}{L_{c}^{2}}\right)\;\frac{\partial N^{NStressG}\left(0,t\right)}{\partial x}$$

(A17) $$\frac{\partial }{\partial x}\;\left(\frac{\partial u\left(L,t\right)}{\partial x}+\frac{1}{2}{\left(\frac{\partial w\left(L,t\right)}{\partial x}\right)}^{2}\right)+\frac{1}{L_{c}}\left(\frac{\partial u\left(L,t\right)}{\partial x}+\frac{1}{2}{\left(\frac{\partial w\left(L,t\right)}{\partial x}\right)}^{2}\right)=\frac{1}{A_{E}}\frac{\xi_{1}}{L_{c}}N^{NstressG}\left(L,t\right)+\frac{1}{A_{E}}\left(\xi_{1}+\frac{L_{l}^{2}}{L_{c}^{2}}\right)\frac{\partial N^{NstressG}\left(L,t\right)}{\partial x}\;$$

(A18) $$-\frac{\partial^{3}w}{\partial x^{3}}\left(0,t\right)+\frac{1}{L_{c}}\frac{\partial^{2}w}{\partial x^{2}}\left(0,t\right)=-\frac{1}{I_{E}}\frac{\xi_{1}}{L_{c}}M^{NstressG}\left(0,t\right)+\frac{1}{I_{E}}\left(\xi_{1}+\frac{L_{l}^{2}}{L_{c}^{2}}\right)\frac{\partial M^{NstressG}\left(0,t\right)}{\partial x}$$

(A19) $$-\frac{\partial^{3}w}{\partial x^{3}}\left(L,t\right)-\frac{1}{L_{c}}\frac{\partial^{2}w}{\partial x^{2}}\left(L,t\right)=\frac{1}{I_{E}}\frac{\xi_{1}}{L_{c}}M^{NstressG}\left(L,t\right)+\frac{1}{I_{E}}\left(\xi_{1}+\frac{L_{l}^{2}}{L_{c}^{2}}\right)\frac{\partial M^{NstressG}\left(L,t\right)}{\partial x}$$

Furthermore, if in Equation (A7) we neglect the axial inertia term, $A_{\rho }\frac{\partial^{2}u}{\partial t^{2}}$, we obtain

(A20) $$N^{NstressG}\left(x,t\right)=A_{E}\left(\varepsilon^{\left(vK\right)}-L_{c}^{2}\frac{\partial^{2}}{\partial x^{2}}\;\varepsilon^{\left(vK\right)}\right)=A_{E}\left(\left(\frac{\partial u}{\partial x}+\frac{1}{2}{\left(\frac{\partial w}{\partial x}\right)}^{2}\right)-L_{c}^{2}\;\frac{\partial^{2}}{\partial x^{2}}\;\left(\frac{\partial u}{\partial x}+\frac{1}{2}{\left(\frac{\partial w}{\partial x}\right)}^{2}\right)\right)=\hat{N}$$

wherein $\hat{N}$ is a constant.

Note that, for a nano-beam with immovable ends ($u{|}_{x=0\;}$= $u{|}_{x=L\;}$= 0 and $w{|}_{x=0\;}$= $w{|}_{x=L\;}$= 0), by integrating both sides of Equation (A20) over the domain [0, L] yields to the following expression

(A21) $$\hat{N}=\frac{A_{E}}{L}\;\int_{0}^{L}\left(\frac{1}{2}{\left(\frac{\partial w\left(x,t\right)}{\partial x}\right)}^{2}-L_{c}^{2}\;\frac{\partial^{2}}{\partial x^{2}}\;\left(\frac{1}{2}{\left(\frac{\partial w\left(x,t\right)}{\partial x}\right)}^{2}\right)\right)dx$$

which coincides with the “mid-plane stretching effect” introduced in [45].

Based on this assumption, from Equation (A12), it follows

(A22) $$-I_{E}\;\frac{\partial^{4}w\left(x,t\right)}{\partial x^{4}}+I_{E}L_{c}^{2}\frac{\partial^{6}w\left(x,t\right)}{\partial x^{6}}+\left(\xi_{1}+\frac{L_{l}^{2}}{L_{c}^{2}}\right)\frac{\partial^{2}}{\partial x^{2}}\left(A_{\rho }\frac{\partial^{2}w\left(x,t\right)}{\partial t^{2}}-I_{\rho }\;\frac{\partial^{4}w\left(x,t\right)}{\partial x^{2}\;\partial t^{2}}-\frac{\partial }{\partial x}\left(\hat{N}\frac{\partial w\left(x,t\right)}{\partial x}\right)+\left(N^{T}+N^{C}\right)\frac{\partial^{2}w\left(x,t\right)}{\partial x^{2}}\right)\;=\left(A_{\rho }\frac{\partial^{2}w\left(x,t\right)}{\partial t^{2}}-I_{\rho }\;\frac{\partial^{4}w\left(x,t\right)}{\partial x^{2}\;\partial t^{2}}-\frac{\partial }{\partial x}\left(\hat{N}\frac{\partial w\left(x,t\right)}{\partial x}\right)+\left(N^{T}+N^{C}\right)\frac{\partial^{2}w\left(x,t\right)}{\partial x^{2}}\right)\;$$

Now, by substituting Equation (A21) into Equation (A22), we obtain

(A23) $$\begin{array}{cc} -I_{E}\frac{\partial^{4}w\left(x,t\right)}{\partial x^{4}}+I_{E}L_{c}^{2}\frac{\partial^{6}w\left(x,t\right)}{\partial x^{6}}\\ & +L_{c}^{2}(\xi_{1}+\frac{L_{l}^{2}}{L_{c}^{2}})\frac{\partial^{2}}{\partial x^{2}}\left(A_{\rho }\frac{\partial^{2}w\left(x,t\right)}{\partial t^{2}}-I_{\rho }\;\frac{\partial^{4}w\left(x,t\right)}{\partial x^{2}\;\partial t^{2}}-\frac{\partial }{\partial x}\left(\left(\frac{A_{E}}{L}\;\int_{0}^{L}\left(\frac{1}{2}{\left(\frac{\partial w\left(x,t\right)}{\partial x}\right)}^{2}-L_{c}^{2}\;\frac{\partial^{2}}{\partial x^{2}}\;\left(\frac{1}{2}{\left(\frac{\partial w\left(x,t\right)}{\partial x}\right)}^{2}\right)\right)dx\right)\frac{\partial w\left(x,t\right)}{\partial x}\right)+\left(N^{T}+N^{C}\right)\frac{\partial^{2}w\left(x,t\right)}{\partial x^{2}}\right)\\ & =\left(A_{\rho }\frac{\partial^{2}w\left(x,t\right)}{\partial t^{2}}-I_{\rho }\;\frac{\partial^{4}w\left(x,t\right)}{\partial x^{2}\;\partial t^{2}}-\frac{\partial }{\partial x}\left(\left(\frac{A_{E}}{L}\;\int_{0}^{L}\left(\frac{1}{2}{\left(\frac{\partial w\left(x,t\right)}{\partial x}\right)}^{2}-L_{c}^{2}\;\frac{\partial^{2}}{\partial x^{2}}\;\left(\frac{1}{2}{\left(\frac{\partial w\left(x,t\right)}{\partial x}\right)}^{2}\right)\right)dx\right)\frac{\partial w\left(x,t\right)}{\partial x}\right)+\left(N^{T}+N^{C}\right)\frac{\partial^{2}w\left(x,t\right)}{\partial x^{2}}\right)\; \end{array}$$

which describes the nonlinear transverse free vibrations of nano-beams in a hygrothermal environment.
